# Knowledge, Practices, and Attitudes Towards Silver Diamine Fluoride Therapy Among Dentists and Students in Southeastern Spain

**DOI:** 10.3390/dj13010020

**Published:** 2025-01-02

**Authors:** Clara Serna-Muñoz, Marina Lucas-Porras, Yolanda Martínez-Beneyto, Amparo Pérez-Silva, Francisco Javier Ibañez-López, Antonio José Ortiz-Ruiz

**Affiliations:** 1 Department of Integrated Pediatric Dentistry, Faculty of Medicine-Dentistry, University of Murcia (Spain), Hospital Morales Meseguer, 2a Planta, C/Marqués de los Vélez, s/n, 30007 Murcia, Spain; clara.serna@um.es (C.S.-M.); marina.lucasp@um.es (M.L.-P.); amparo.perez@um.es (A.P.-S.); ajortiz@um.es (A.J.O.-R.); 2 Department of Preventive Dentistry, Faculty of Medicine-Dentistry, University of Murcia (Spain), Hospital Morales Meseguer, 2a Planta, C/Marqués de los Vélez, s/n, 30007 Murcia, Spain; 3 Maths Department, Didactics of Mathematics Area, Faculty of Education, Campus de Espinardo, University of Murcia, 3a Planta, 30007 Murcia, Spain; fjil@um.es

**Keywords:** silver diamine fluoride, dental caries, survey, knowledge, attitude

## Abstract

**Background/Objectives:** The use of silver diamine fluoride (SDF) has increased in recent years for the management of caries lesions in children and adults. The aim of this study is to determine the level of knowledge and the attitude of Spanish dentists (GDPs) and final-year dental students (DSs) regarding the use of SDF. **Methods:** A cross-sectional survey (questionnaire) was carried out aimed at final-year dental students (DSs) (n = 43) and registered dentists (GDPs) (n = 1050) in the autonomous community of the region of Murcia (Spain). **Results:** the response rates were GDPs 7.7% (n = 81) and DSs 84.5% (n = 38). Only 20.98% of GDP respondents reported having been trained on SDF versus 100% of DSs. Significant differences were observed between the groups (*p* < 0.05). While 94.7% of the students were aware of the indications for the use of SDF, only 56.8% of the general dentists reported it. Similarly, for hypersensitivity treatment, 71.1% of the students were informed versus 40.7% of the general dentists, and indications for paediatric patients, 100% for the DS group and 59.3% in GDPs. In adult patients, indications vary from GDPs’ (50%) to DSs’ (25.9%) responses. About 94.7% of DSs know the advantages of use and only 50.6% of GDPs. Both groups showed reluctance to use SDF in esthetic zones, with greater acceptance in non-esthetic areas (*p* < 0.05). In practice, fewer GDPs (27.16%) and DSs (23.68%) had applied SDF, reflecting a gap between knowledge and implementation. **Conclusions:** Dental students had a significantly higher level of knowledge, a situation that evidences the high level of education and training in the curricular guides of the universities.

## 1. Introduction

Dental caries is a non-communicable and preventable disease. It is a destruction of the calcified structures of the tooth due to the action of acids generated by the bacteria present in dental plaque, which are capable of producing demineralisation [1]. Under normal conditions, this demineralisation is in equilibrium with remineralisation. However, when the environment is not favourable, there is an imbalance, and the rate of remineralisation does not compensate for the rate of demineralisation. This is a condition that can affect general health and quality of life, because it can become painful, and it can influence the development of new lesions, school performance, children’s rest, etc. [2].

Early childhood caries has a prevalence of 60–90% worldwide. At the European level, statistics show that 61% of children aged 6–12 years have at least one tooth affected by caries [3].

Currently, the most commonly used therapy for the treatment and removal of dental caries in children is called minimal intervention dentistry. The aim of this technique is to preserve as much tooth structure as possible by determining the caries risk of each patient, recognising early stages of non-cavitated caries and identifying whether it is an active or an inactive lesion, in order to implement different protocols for action [4].

In addition to toothpastes, more effective methods for preventing and reversing caries lesions [5] include materials that are applied in the healthcare setting such as fluoride varnishes, fluoride-releasing pit and fissure sealants, pure or resin-modified glass ionomers, or silver diamine fluoride (SDF) [2].

Although the use of silver diamine fluoride is increasing, this material was developed in Japan in the 1960s by Misuho Nishino. Subsequently, in 1972, the indications and uses of SDF were published [6].

SDF was defined by the Food and Drug Administration in the United States in 2014 for managing dentine hypersensitivity [7]. Since then, there has been growing interest in its “off-label” use for managing carious lesions, especially in children [8]. SDF at a concentration of 38% and applied every 6 months or yearly, is effective in stopping cavitated carious lesions in primary teeth [9]. Published evidence supports its use in primary dentition with children at high caries risk, patients with disabilities, medically compromised patients, as well as to reduce sensitivity, root and coronal caries in adult patients, and patients requiring numerous dental visits [3,10].

The World Health Organization (WHO) has recently (2021) included SDF among the essential medicines for the management of dental caries for both adults and children [11].

Among the main advantages of its use, it is easy to apply, fast, requires little patient collaboration, and is a non-invasive technique that does not require anaesthesia or rotating instruments [12], and among its main disadvantages is dental staining [3].

Studies have been published that focus on determining knowledge and attitudes about silver diamine fluoride in countries such as Japan, the USA, Saudi Arabia, the Netherlands, etc. [13,14,15,16,17]. However, no studies have been described that relate the level of knowledge of working dentists to future dentists (dental students), a situation that could identify a change in future clinical practice related to the level of knowledge of students.

The main objective of this study is to assess the current knowledge, experience, and attitude of dentists in southeastern Spain (region of Murcia) and final-year dental students at the University of Murcia regarding the use of silver diamine fluoride in the management of caries lesions.

## 2. Materials and Methods

### 2.1. Sample and Procedures

The methodology for obtaining the necessary data for this cross-sectional study was based on a survey. The data were processed in accordance with the Law on the Protection of Personal Data and Guarantee of Digital Rights (Organic Law 3/2018).

The survey was designed in the survey platform of the University of Murcia to generate a link accessible to all participants. The link was as follows: https://encuestas.um.es/encuestas/sdf.cc (accessed on 27 February 2023).

The questionnaire was addressed to dentists (GDPs) in the region of Murcia (Spain) (n = 1050) and fifth-year dental students (DSs) at the University of Murcia (n = 43), informing all of them that the survey would be anonymous and voluntary. It was sent on 27 February 2023, and an email was sent 2 weeks after the initial distribution. The survey was online for 2 months.

A pilot version of the questionnaire was tested by five professors of the dentistry faculty and five undergraduate students, University of Murcia, to ensure the questions had been correctly prepared, were easily understandable, and did not entail a prolonged response time.

The questionnaire was divided into three main sections (31 questions) following the review of questionnaires published in recent articles [13,14,15,17].

The first block corresponded to socio-demographic data, and the second section addressed knowledge and acceptance of using SDF. The third section referred to its use for different patient situations and clinical lesions that can be encountered. The Likert scale was used with a ranged from 1 to 5, where 1 was strongly disagree and 5 denoted strongly agree.

The reliability and consistency of this instrument was assessed using several indices. Cronbach’s alpha yielded a value of α = 0.929, considered excellent [18]. In addition, the composite reliability coefficient and the average variance extracted (AVE) were calculated, obtaining values of 0.932 and 0.408, considered excellent [19]. Finally, an Omega coefficient of 0.955 was obtained, also considered excellent [20].

### 2.2. Data Analysis

Study data were processed and analysed using the R Statistical package. A simple frequency distribution was made. Non-parametric tests were applied, as these are the most robust tests for ordinal data. Specifically, the Mann–Whitney U test was used for independent variables with two levels of response and the Kruskal–Wallis test (non-parametric ANOVA) for variables with more than two levels (*p*-value less than 0.05 and significance level α = 0.05). For the post-hoc, the pairwise Wilcoxon rank sum test with Bonferroni correction was performed.

## 3. Results

### 3.1. Participants

The overall response rate was 84.4% (n = 38) DSs and 7.7% (n = 81) GDPs. In total, 119 responses were obtained.

Regarding age range, it varied from 22 to 66, with a total mean age of 42.86 (SD 12.02) years for GDPs and 22.71 (SD 1.56) for students. In terms of gender, 68.1% of the responses were female and 31.9% were male.

Most of the dentists were in private practice (91.35%). The members completed their studies between 1980 and 2022, the most frequent year being 2001 with 7.6% of the responses, with an average of 22 years of work experience.

### 3.2. Descriptive Data

Only 20.98% of the GDPs admitted having received training on SDF in their academic stage, compared to 100% of the students. Only 27.16% of the GDPs had ever used SDF and 23.68% of the final-year students.

In this regard, the majority of DSs agreed/strongly agreed (94.7%) that they were aware of the indications for the use of SDF, compared to 56.8% of GDPs (*p* < 0.05). A total of 71.1% of DSs agreed/strongly agreed that they were aware of its use to treat hypersensitivity, compared to 40.7% of GDPs (*p* < 0.05). For use in paediatric dentistry, to treat lesions in the primary dentition, 100% of the SDs confirmed that they knew how to use this material, compared to 59.3% of GDPs, and 50% in the case of permanent teeth, a value that drops to 25.9% for GDPs (*p* < 0.05). Regarding the advantages of its use compared to conventional treatments, 94.7% of DSs were aware of it, decreasing to 50.6% for GDPs (*p* < 0.05). Similar response values were obtained for the knowledge of the disadvantages of the use of SDF, 89.5% DSs and 50.6% GDPs (*p* < 0.05). (Table 1)

Table 2 describes the degree of agreement (codes 4 and 5, Likert scale) obtained in both groups regarding the use of SDF for different clinical situations. It is worth noting that 89.5% of the students agreed/strongly agreed with the use of SDF to stop enamel caries, a value that drops to 59.3% in the case of GDPs, with statistically significant differences being observed (*p* < 0.05).

In the case of its use for arresting caries in dentine, 78.9% of the students agreed/strongly agreed that such a material could be used, a value that dropped to 66.7% in the group of dentists. On the assumption of arresting of cavitated root caries by SDF, the value drops to 47.4% of DSs and 43.2% GDPs. Almost half of DSs (42.1%) agreed/strongly agreed that carious dentine removal should be carried out prior to the application of SDF compared to 19.8% of GDPs.

Finally, 84.2% of DSs identified the use of SDF as a treatment alternative when lesions cannot be restored conventionally in a single appointment, a value that was reduced to 61.7% in the case of GDPs (*p* < 0.05).

Regarding indications on the type of patient suitable for using SDF, it should be noted that 100% of DSs answered that they agreed/fully agreed to use it in paediatric patients with behavioural management difficulties, compared to 77.8% of GDPs (*p* < 0.05), followed by patients who cannot financially afford expensive restorative treatments, with 94.7% of students and 55.6% of GDPs (*p* < 0.05). No significant differences were found between the two study groups over concerns to medically compromised patients, patients with dental anxiety, patients who are receiving or have received radio/chemotherapy, patients taking bisphosphonates, patients requiring treatment under general anaesthesia, or patients with microstomia (Table 3).

Finally, when SDF is used in aesthetic areas, the majority of respondents say they do not agree with its use in both primary (68.9%) and permanent (81.5%) dentition (Table 4). These values are inverted in the case of use in non-aesthetic areas, where both in primary (80.7%) and permanent (64.71%) dentition, its use would be more accepted among professionals (*p* < 0.05) (Table 4).

## 4. Discussion

Since silver diamine fluoride has not been widely used by Spanish dentists in recent years, this is the first study to examine the knowledge and attitudes of dentists and dental students in Spain.

The use of SDF has not been very popular in Spain, but its use is increasing since it has been introduced as a preventive material in the public dental services of the national health system, as a consequence of the enforcement of the 2023–2030 objectives.

In the degree in Dentistry at the University of Murcia, internships with paediatric patients are carried out in the final year. It is in this year that students receive the most information about the handling of silver diamine fluoride and put into practice the theoretical knowledge learnt in previous years. Moreover, at this stage, the status of professional vs. student is only differentiated for a few months. The knowledge students have that last year should be what sets the attitudes and clinical skills. For this reason, final-year students were selected.

In general, a low degree of training on the management of SDF has been observed among dental professionals, but not among students. In this regard, it was found that overall, only 53.8% of the participants had received academic training on SDF, including 100% of the students; similar values (94.2%) were described by Dang et al. [14] in the USA for this group of students. However, the training drops to 20.98% of the GDPs in our study. A study published by Antonioni et al. [16] (also in the USA) described much lower values (3%) for paediatric dentists. In Spain, the curriculum of cariology has recently been published [17,21] ith the participation of representatives from both public and private Spanish universities. This breakthrough has made it possible to unify criteria in terms of teaching competencies on the management of dental caries, including the management of SDF. Due to this curricular incorporation, the results in terms of knowledge of SDF indications are 100% of Spanish university students.

Only 26.1% of the participants in this study had ever used SDF, of which 29% were students. Also in the studies by Alajlan et al. [15] and Dang et al. [14], the percentage of participants who use it is low, as well as in the survey by Antonioni et al. in the USA (98% of respondents had not used it frequently in their academic training) [16]. Schroë et al. in Denmark also described that 84% of their respondents were aware of the uses of SDF [17]. SDF was first introduced in Japan for use in dentistry, and as such, has the highest usage rate in the country, even higher than the country’s own university students [13]. A possible justification for the low rate of use in our environment could be the mainly aesthetic disadvantages that exist with its use [7] and the lack of academic training.

In order for this material to be used, it is important that the main advantages and disadvantages of its use are known. In our study, 64.71% of the participants knew the advantages of SDF and 63.03% the disadvantages. However, the level of knowledge is always higher in the group of students compared to GDPs (*p* < 0.05). The values obtained coincide with those described by Antonioni et al. in a study carried out in paediatric dentists in the USA [16].

The effectiveness of the use of SDF as a desensitiser agent has been described [10]. In our study, 71.1% of SDs were aware of this treatment therapy compared to 40.7% of GDPs, values slightly lower than those described in the USA by paediatric dentists (51%) [16].

The use of SDF for permanent dentition can reduce costs and increase benefits, especially in institutionalised, hospitalised, and dependent patients with limited financial resources and mobility [14]. It has been recommended for patients with dementia and xerostomia [13]. In our study, the indication for SDF for cavitated root caries was 43.2% for GDPs and 47.4% for students. However, there are studies in Japan where these values are as high as 87% [13]. In Japan, the rate in the geriatric population is higher than in the paediatric population; however, in more Western countries, its use is still more associated with the primary dentition and less with the permanent dentition, possibly due to staining.

In 2002, it was described that the removal of soft dentine prior to SDF application was not necessary [22]. In our study, it was shown that 42.86% agreed and/or strongly agreed that this step is necessary, slightly lower values than those published in the Antonioni et al. study (59%) [16]. The removal of infected dentine versus non-removal has not demonstrated greater effectiveness of the fluoride materials used in caries lesions, which is why it is concluded that it is not necessary to remove infected dentine. Thus, SDF treatment would also have a shorter chair time [22].

One of the main drawbacks with the use of SDF is the staining that occurs [8]. The black staining, particularly on the anterior front teeth, is unaesthetic, causing patient dissatisfaction. A recent study has been published [23], which indicates that the use of SDF for posterior teeth is widely accepted by both professionals and patients, but in anterior teeth where the staining is unsightly, patients do not accept it. This has been found to be especially related to male patients of high economic status. In addition, it has been reported that when parents have prior information about its use, there is much less resistance to its use; for example, information through official websites of scientific societies [17].

The majority of the participants (94.7%) agreed that the ideal areas for the application of SDF are the non-aesthetic areas of both dentitions; however, they were more inclined to use it in the primary dentition. This could be explained by one of the disadvantages of the material, which is the dark staining of the surface where it is applied. In the study by Schroë et al. [17], 0.62% of paediatric dentists and 37% of general dentists stated that they would use SDF in posterior regions and not in anterior regions of primary teeth. In contrast, Chai et al. found that 88% of their participants would use it in anterior areas of deciduous dentition [13].

Due to the unsightly staining caused by SDF, new studies are emerging, such as that of Almuqrin et al. [24], which concludes that there are several methods to reduce such staining, such as silver nanoparticles (AgNP), silver fluoride, or hydroxyapatite nanoparticles. Since staining results from oxidation processes, adding antioxidant components such as selenium nanoparticles can also help.

However, in deciduous dentition, this disadvantage is temporary, as it will last until the tooth exfoliates or is extracted. This is why it is not usually used in permanent dentition, especially in the coronal area; however, caries in root areas is a good indication to apply SDF and thus slow down its progression [25]. In addition, combined with glass ionomer when a filling is necessary in this type of caries, it can increase the effectiveness of ionomer [26]. This is because together they act synergistically, as the fluoride in SDF and glass ionomer will react with the calcium and phosphate in the odontoblastic processes, creating a caries-resistant base under the ionomer. Also, where carious dentin is present, fluorapatite will form, which is resistant to the action of acids [27].

However, although many parents do not tolerate staining well, they do accept the use of SDF to avoid much more invasive treatments, where in most cases, general anaesthesia is necessary [28]. In our study, 86.8% of the students and 63% of the dentists agreed to use SDF as an alternative when they want to postpone treatment under general anaesthesia, results slightly higher than those published by Antonioni et al. (50%) [16] and by Alajlan et al., in which only 36.69% of the participants agree [15]. General anaesthesia in dentistry is used when the management of the patient is very complicated, and the patient has many carious lesions to treat. One drawback is that general anaesthesia will not reduce the child’s anxiety, which is the main factor for poor management. This is why treatment alternatives such as minimally invasive dentistry (IMD), which includes techniques such as atraumatic restorative treatment (ART) and the use of silver diamine fluoride, have emerged [29].

Although the ART technique does not include the use of SDF, the SMART technique (silver-modified atraumatic restorative technique) has been created, which involves removing carious dentine with spoons and manual elements, applying 38% SDF to stop the caries and finishing with the application of a glass ionomer, making the filling and at the same time taking advantage of its remineralising properties [27]. Abdellatif et al. concluded that the ART technique and SDF are effective as a treatment for caries arrest in the primary dentition. However, if the patient requires little chair time or have the necessary materials are unavailable, SDF will be the treatment of choice, as it is quicker [30].

Moreover, in arresting caries, SDF therapy is non-invasive and painless. It helps to reduce patients’ dental anxiety and fear of dental care. A clinical study found that only 4% of young children with caries were uncooperative during treatment and could not receive SDF therapy [31]. In our study, 86.9% of the students strongly agreed that the use of SDF allows a control of the patient’s anxiety, compared to 69.1% of the GDPs, values slightly lower (81%) than those published in the USA [16] or even higher than those published in Saudi Arabia (55.4%) [15]. Regarding the indication for paediatric patients with misbehaviour in consultation, it should be noted that a statistically significant difference has been observed between the response of the students (100%) compared to 77.8% of the GDPs, values very similar to those published in the study by Antonioni et al. [16].

In addition to this indication for the use of SDF in uncooperative or anxious paediatric patients, the American Academy of Paediatric Dentistry guidelines [28] indicates its use in medically compromised patients, patients with dental anxiety, those taking bisphosphonates, those receiving radiotherapy or chemotherapy treatment, and those with microstomia [14,15,16]. In our work, slightly lower values have been obtained than in the rest of the studies [14,15,16] in GDPs but much higher for the group of Spanish dental students.

In this study, 94.7% of the students and 55.6% of the dentists agreed that SDF is a treatment for patients who cannot afford conventional restorative treatments. However, in the study by Alajlan et al., only 39.21% of the participants accepted its low cost as an advantage [15], similar values (34%) to those described in the USA [16].

Despite existing international guidelines for the management of dental caries in both children and adults, where the use of fluorides is the most effective tool to prevent and control even very deep lesions, there are published studies [8] where the reluctance of parents to use fluoride products in children is evident. This, together with the staining of treated teeth, makes it a requirement to obtain written informed consent from parents or guardians.

Among the main limitations of the study, the low response rate for registered dentists is particularly noteworthy. This could be due to the fact that the surveys conducted via e-mail make the response rate low due to the saturation of e-mails received [32]. However, it could also be explained from the point of view that in Spain, the use of SDF among dentists is very low, and the title of the survey could have led those who did not participate to do so because of the title or subject of the survey.

## 5. Conclusions

The results observed in the study indicate that new generations of Spanish dentists are more knowledgeable in SDF than current GDPs. These results have also been observed in other countries such as the USA, where the curricular guides in cariology have been updated [16].

It would be necessary to carry out this study in other Spanish regions, both universities and registered dentists, in order to extrapolate data that would be representative of Spanish dental care activity. However, this pioneering work highlights the possible gaps that could be detected, serving as a reference for future strategies to provide more training for general dentists in the management of caries.

As SDF has been incorporated into public dental services, GDPs will need to update their training through refresher courses on minimally invasive materials.

## Figures and Tables

**Table 1:** Participants’ responses regarding SDF knowledge.

Likert Scale (1–5) for Values “Agree” (4) or “Strongly Agree” (5)	Both Groups	Data for Dental Students		Data for GDPs		*p*-Value *
	(%)	(%)	Mean ** (SD)	(%)	Mean ** (SD)	
What is SDF used for in dentistry?	68.91%	94.7%	4.47 (0.6)	56.8%	3.37 (1.38)	0.000
Use of SDF for the treatment of hypersensitivity	50.42%	71.1%	4 (0.77)	40.7%	3.10 (1.29)	0.000
Use of SDF for the treatment of dental caries in paediatric dentistry	72.27%	100%	4.66 (0.48)	59.3%	3.53 (1.38)	0.000
Use of SDF for the treatment of caries in adult patients	33.61%	50%	3.45 (1.03)	25.9%	2.69 (1.26)	0.002
Advantages that SDF treatment can have over conventional dental treatments	64.71%	94.7%	4.53 (0.6)	50.6%	3.27 (1.22)	0.000
Possible disadvantages of using SDF	63.03%	89.5%	4.21 (0.84)	50.6%	3.23 (1.27)	0.000

* Significant difference if *p* < 0.05. The *p*-values represented by 0.000 are values very close to 0. ** Mean and standard deviation values for total responses, 0–5 Likert scale.

**Table 2:** Level of knowledge of clinical indications for the use of SDF for dental treatment.

Likert Scale (1–5) for Values “Agree” (4) or “Strongly Agree” (5)	Total Groups	Data for Dental Students		Data for GDPs		*p*-Value *
	(%)	(%)	Mean ** (SD)	(%)	Mean ** (SD)	
SDF can be used to stop lesions in enamel.	68.91%	89.5%	4.21 (0.78)	59.3%	3.57 (1.09)	0.001
SDF can be used to stop lesions in dentine.	70.59%	78.9%	4.03 (1.05)	66.7%	3.80 (1.02)	0.160
SDF can be used to stop cavitated root caries lesions.	44.54%	47.4%	3.34 (1.07)	43.2%	3.31 (1.23)	0.988
Infected dentine must be removed prior to SDF application.	26.89%	42.1%	3.03 (1.37)	19.8%	2.60 (1.17)	0.095
SDF is a good treatment when lesions cannot be restored in one appointment.	68.91%	84.2%	4.21 (1.02)	61.7%	3.78 (1.2)	0.050

* Significant difference if *p* < 0.05. The *p*-values represented by 0.000 are values very close to 0. ** Mean and standard deviation values for total responses, 0–5 Likert scale.

**Table 3:** Level of knowledge of clinical indications for the use of SDF according to patient characteristics.

Likert Scale (1–5) for Values “Agree” (4) or “Strongly Agree” (5)	Both Groups	Data for Dental Students		Data for GDPs		*p*-Value *
	(%)	(%)	Mean ** (SD)	(%)	Mean ** (SD)	
Paediatric patients with behavioural management difficulties	84.87%	100%	4.58 (0.5)	77.8%	4.1 (1.04)	0.021
Medically compromised patients	54.62%	63.2%	3.74 (0.79)	50.6%	3.51 (1.24)	0.464
Patients with dental anxiety	74.79%	86.8%	4.26 (0.76)	69.1%	3.86 (1.08)	0.069
Patients who are receiving or have received radiotherapy/chemotherapy	57.14%	65.8%	3.82 (0.77)	53.1%	3.6 (1.13)	0.432
Patients taking bisphosphonates	43.7%	50%	3.58 (0.72)	40.7%	3.42 (1.07)	0.429
Patients requiring treatment under general anaesthesia, in order to postpone it	70.59%	86.8%	4.11(0.92)	63%	3.81 (1.1)	0.143
Patients with microstomia	42.02%	47.4%	3.55 (0.89)	39.5%	3.46 (0.95)	0.529
Patients who cannot financially afford restorative treatment	68.07%	94.7%	4.39 (0.59)	55.6%	3.51 (1.29)	0.000

* Note: significant difference if *p* < 0.05. *p* values represented by 0.000 are values very close to 0. ** Mean and standard deviation values for total responses, 0–5 Likert scale.

**Table 4:** Attitudes towards clinical indications for the use of silver diamine fluoride according to caries topography.

Likert Scale (1–5) for Values “Disagree” (2) or “Strongly Disagree” (1)	Both Groups	Data for Dental Students		Data for GDPs		*p*-Value *
	(%)	(%)	Mean ** (SD)	(%)	Mean ** (SD)	
Aesthetic zone of primary teeth	68.91%	73.7%	2.13 (1.19)	66.7%	2.21 (1.1)	0.541
Aesthetic zone of permanent teeth	81.51%	81.6%	1.89 (1.13)	81.5%	1.72 (0.83)	0.696
Likert scale (1–5) for values “agree” (4) or “strongly agree” (5)						
Non-aesthetic area of primary teeth	80.67%	94.7%	4.63 (0.75)	74.1%	4 (1.12)	0.001
Non-aesthetic area of permanent teeth	64.71%	71.1%	3.95 (1.09)	61.7%	3.65 (1.06)	0.125

* Significant difference if *p* < 0.05. The *p*-values represented by 0.000 are values very close to 0. ** Mean and standard deviation values for total responses, 0–5 Likert scale.

## Data Availability

The data presented are available upon request to the corresponding author.
